# Estimating the impact of pill burden on health utilities in hemodialysis therapy

**DOI:** 10.1038/s41598-025-19346-3

**Published:** 2025-10-09

**Authors:** Hiroo Shimoda, Masatomo Taniguchi, Suguru Yamamoto, Ataru Igarashi, Shin Tokunaga, Keigo Hanada, Naoki Tashiro, Tatsunori Murata, Shinji Asada

**Affiliations:** 1 Medical Affairs Department, Kyowa Kirin Co., Ltd, Chiyoda-ku, Tokyo, Japan; 2 Division of Nephrology, Fukuoka Renal Clinic, Fukuoka-city, Fukuoka Japan; 3 Division of Clinical Nephrology and Rheumatology, Niigata University Graduate School of Medical and Dental Science, Niigata-city, Niigata Japan; 4 Department of Health Policy and Public Health, Graduate School of Pharmaceutical Sciences, The University of Tokyo, Tokyo, Japan; 5 CRECON Medical Assessment Inc, Shibuya-ku, Tokyo, Japan

**Keywords:** Cost of illness, Health status, Quality-adjusted life years, Renal dialysis, Nephrology, Quality of life

## Abstract

Patients undergoing hemodialysis often require numerous medications because of comorbidities including hyperphosphatemia, diabetes, and anemia, coupled with water restrictions. Reducing pill burden may alleviate psychological stress and improve quality of life (QOL). However, standardized instruments such as the EuroQOL 5 Dimensions inadequately measure these effects. We investigated the impact of pill burden on utility scores using a vignette-based time trade-off (TTO) method. Members of the general population were interviewed in person using vignettes representing seven health states with varying daily pill burdens (2, 3, 6, 9, 12, 24, and 36 tablets) in the context of hemodialysis patients, including disease characteristics, symptoms, and daily life restriction. Utility scores were evaluated using the TTO method and the impact of pill burden on QOL was estimated. The mean age (44.5 years) and sex distribution (50.5% male) of the 107 participants closely matched the general population of Japan. Utility scores decreased with higher pill burdens, ranging from 0.373 (0.579) [mean (standard deviation)] for 2 tablets/day to 0.239 (0.568) for 36 tablets/day. Compared with 2 tablets/day, the utility score for 6 tablets/day was significantly lower [mean (standard deviation) difference: 0.046 (0.223), *P* = 0.034, paired t-test)], with the difference showing a more significant as the number of tablets increased. Subgroup analyses by sex and age showed consistent trends with no significant differences between groups. A higher pill burden was associated with lower utility scores, highlighting its negative impact on QOL in patients undergoing hemodialysis. Reducing pill burden may improve QOL and address challenges related to water restrictions.

**Supplementary Information:**

The online version contains supplementary material available at 10.1038/s41598-025-19346-3.

## Introduction

Chronic kidney disease (CKD) is a progressive Loss of kidney function that may result in kidney replacement therapies such as dialysis or kidney transplantation. CKD affects an estimated 843.6 million individuals worldwide, with 3.9 million undergoing renal replacement therapy^1^. CKD is associated with comorbidities such as hypertension, cardiovascular disease, diabetes, anemia, and mineral and bone disorder, making it a leading risk factor for mortality^2^ and a growing cause of death^3,4^.

The presence of multiple comorbidities necessitates a high number of prescribed medications, such as phosphate binders, antihypertensives, and antihyperglycemic drugs^5^. A survey of 700 patients undergoing maintenance hemodialysis in Japan showed an average intake of more than 16 oral medications daily, including up to 36 tablets of phosphate binders^6^. Phosphate binders are prescribed to 92.2% of patients undergoing hemodialysis to lower the serum phosphate level, preventing mineral and bone disorder progression and improving survival^7–10^. Notably, phosphate binder tablets are usually large, which can pose challenges because water intake is often restricted for patients with CKD undergoing hemodialysis. This situation may adversely impact quality of life (QOL). Approximately 20% of patients undergoing hemodialysis reported a mental burden due to the high number or variety of medications^10^. Furthermore, 37.8% of these patients felt that reducing even one tablet would alleviate this mental burden^10^. Therefore, understanding the impact of the number of tablets on QOL in patients undergoing hemodialysis is important for optimizing CKD treatment.

Quantifying QOL is essential for cost-utility analyses in health technology assessments. Standardized, validated preference-based measures such as the EuroQOL 5 Dimensions (EQ-5D) and Health Utilities Index (HUI) 2/3^11,12^ are widely used but may inadequately capture specific aspects of treatment or changes in QOL^13^. The EQ-5D questionnaire contains five domains (mobility, self-care, usual activities, pain/discomfort and anxiety/depression), each with five severity levels (extreme, severe, moderate, some problems and no problems). However, its limited domains make it difficult to evaluate factors related to medication adherence. One previous study^14^ evaluated pain and anxiety associated with low adherence in patients with diabetes using the EQ-5D, but did not directly evaluate low adherence itself. Other studies have used vignette-based methods to evaluate the utility scores related to pill burden in hepatitis C treatment^15^ and HIV antiretroviral therapy^16^. These studies indicated higher pill burden were associated with lower health utilities and suggest the relevance of evaluating the impact of adherence-related factors such as pill burden on utility scores using vignette-based methods, but none have done so in the context of patients undergoing dialysis. Vignette-based methods, which describe hypothetical health states, provide a flexible approach for assessing such impacts^11,12^. Vignettes, also known as health scenarios, describe hypothetical health states that allow the general population to imagine and valuate the state using methods such as time trade-off (TTO) or standard gamble.

This study employed a vignette-based TTO method to evaluate exploratively how the pill burden impacts utility scores for QOL in patients undergoing hemodialysis.

## Materials and methods

### Study participants

This study was conducted in accordance with the Japanese Ethical Guidelines for Medical and Health Research Involving Human Subjects, which complies with the Declaration of Helsinki. The study protocol was approved by an independent institutional review board of the Public Health Research Foundation (Tokyo, Japan) (Approval number: 24C0004). Informed consent was obtained from all individual participants included in the study. Participants were recruited between March and August 2024 by external market research companies (ASMARQ Co., Ltd., Tokyo, Japan for participants with CKD undergoing hemodialysis; INTAGE Research Inc., Tokyo, Japan for general participants). The study was divided into a pilot survey and main survey. The pilot survey included both general participants and participants with CKD undergoing hemodialysis, while the main survey involved only general participants. The inclusion criteria for general participants were an age of ≥ 20 years, residence in Japan, the ability to understand and follow the study procedures, and willingness to provide consent at the time of recruitment. The inclusion criterion for participants with CKD undergoing hemodialysis was being on maintenance hemodialysis at the time of recruitment. Individuals who could not provide written or online consent or who were unable to complete the interview procedures independently were excluded from both groups.

### Vignette development

This vignette-based TTO study was conducted following the recommendations of Matza et al.^13^. The vignettes were developed and refined using data from previous studies, clinical information, and input from patients with CKD undergoing hemodialysis and medical experts regarding hemodialysis treatment^17–19^. The scenarios depicted patients with CKD undergoing hemodialysis with seven different health states based on the daily number of tablets of concomitant medication. To isolate the effect of pill burden on utility scores, other aspects of the health states—such as the disease itself, associated symptoms, and daily life restrictions (including water intake limitations)—were held constant across all scenarios. The vignettes detailed the number of doses per day, the number of tablets per dose, and the water intake allowed per day and required per dose across the seven health states: 2, 3, 6, 9, 12, 24, and 36 tablets/day.

A pilot study was conducted to ensure that the vignettes were easily understandable and to identify and address potential issues in the interview procedures. Ten general participants and 10 patients with CKD participated in the pilot study and went through the interview procedure. Feedback from the participants was reviewed with medical experts, and the vignettes and interview procedures were finalized for use in the main survey (Tables 1 and 2). We focused on creating accurate and easily understandable vignettes by incorporating feedback from participates with CKD undergoing hemodialysis and medical expert in dialysis therapy. This feedback addressed the characteristics of the disease, the real-world context of hemodialysis therapy and its burden, various complications and their management, and the constraints on water intake resulting from impaired kidney function. Based on the results of previous studies^20–22^, the target sample size for the main survey was set at 100 valid interviews from general participants.

**Table 1:** Vignette used in the time trade-off and lead-time trade-off methods: common elements across all seven states.

Item	Description
Disease	- I have kidney disease. I cannot remove excess water, excess salt, and wastes from my body. At the same time, I cannot keep essential substances in my body. - Without appropriate treatment, I may die sooner. - I have to be treated at a hospital 3 times a week for 3 to 4 h each visit, without any break. - I have to take drugs every day to treat comorbidities.
Symptoms	- When the kidney disease advances, I experience symptoms such as swelling, tiredness, and anemia. - I experience heart pounding or shortness of breath when exercising. - I have itchy skin, and it becomes worse by taking a hot water bath.
Restrictions in daily life	- The amount of water I can drink is restricted to 900 mL (per 60 kg body weight), including a glass of water (200 mL) each time I take drugs, to prevent harmful effects on my heart and blood vessels by retaining excess water. - The amount of nutrients and salts in my food must be strictly controlled to prevent harmful effects on my heart and blood vessels.

**Table 2:** Health States: seven health States based on the daily number of tablets of concomitant drugs.

Health state	Description
2 tablets/day	- I take 2 tablets/capsules every day. - I take 1 tablet/capsule at a time, twice a day. - From my 900-mL daily allowance of water, I use 400 mL for taking medicine.
3 tablets/day	- I take 3 tablets/capsules every day. - I take 1 tablet/capsule at a time, three times a day. - From my 900-mL daily allowance of water, I use 600 mL for taking medicine.
6 tablets/day	- I take 6 tablets/capsules every day. - I take 2 tablets/capsules at a time, three times a day. - From my 900-mL daily allowance of water, I use 600 mL for taking medicine.
9 tablets/day	- I take 9 tablets/capsules every day. - I take 3 tablets/capsules at a time, three times a day. - From my 900-mL daily allowance of water, I use 600 mL for taking medicine.
12 tablets/day	- I take 12 tablets/capsules every day. - I take 4 tablets/capsules at a time, three times a day. - From my 900-mL daily allowance of water, I use 600 mL for taking medicine.
24 tablets/day	- I take 24 tablets/capsules every day. - I take 8 tablets/capsules at a time, three times a day. - From my 900-mL daily allowance of water, I use 600 mL for taking medicine.
36 tablets/day	- I take 36 tablets/capsules every day. - I take 12 tablets/capsules at a time, three times a day. - From my 900-mL daily allowance of water, I use 600 mL for taking medicine.

### Interview procedures

Participants with CKD undergoing hemodialysis in the pilot survey were interviewed online via a video conference call, while general participants in both the pilot and main surveys were interviewed in person at a venue in Tokyo, Japan. Ten well-trained interviewers conducted the main survey. Participants were first asked for demographic information and screened based on the inclusion and exclusion criteria. After providing consent (online for participants with CKD and written for general participants on-site), the participants answered four training questions designed to clarify their understanding of life-threatening CKD, learned how to compare two health states, and practiced TTO valuations. The TTO practice included two hypothetical health states: one requiring lifelong wheelchair use and another that was incurable and considered worse than death. The participants then proceeded with the vignette-based TTO valuation for utility scores in CKD, and their current clinical status was recorded at the end.

### Vignette-based TTO valuation for utility scores

Using the finalized vignettes (Tables 1 and 2), utility scores were estimated by either the lead-TTO or TTO methods^23^ (Supplementary Fig. S1). Seven virtual health states based on the daily number of tablets were presented to participants in random order. Participants were first asked to select among three options: (1) die immediately; (2) live 10 years with the health state, then die; or (3) both options are the same.

If participants selected option 1), the health state was considered “worse than dead” and the lead-TTO method was employed. The participants were given three new options: (1) live 10 years in “full health,” then die; (2) live 10 years in “full health” followed by 10 years with the health state, then die; or (3) both options are the same. If participants selected option 1), the question was repeated with reduction of the “full health” period in option 1) to 5 years. The process continued with incremental addition/reduction of the “full health” period by 1 year according to the participant’s choice (the process of implementing the TTO method^24^ until the participant selected option 3) or switched between options 1) and 2). The utility score was calculated as follows (Eq. 1):

1 $${\rm{Utility \:score = x/10-1}}$$

where x represents the “full health” period in option 1) when the participants selected option 3) or the mean of the “full health” periods in option 1 when the participants switched between options 1) and 2).

If participants selected option 2) in the initial question, the health state was considered “better than dead” and the TTO method was employed. The participants were given three new options: (1) live 5 years in “full health,” then die; (2) live 10 years with the health state, then die; or (3) both options are the same. The process continued using the process of implementing the TTO method, adding/reducing the “full health” period in option 1) until the participant selected option 3) or switched between options 1) and 2). The utility score was calculated as follows (Eq. 2):

2 $${\rm{Utility\: score = x/10}}$$

where x represents the “full health” period in option 1) when the participant selected option 3) or the mean of the “full health” periods in option 1 when the participants switched between options 1) and 2).

If participants selected 3) in the initial question, the utility score was 0.

### Statistical analysis

Patient characteristics were summarized descriptively, with national standard values from Japanese official statistics^24–32^ used as references. Utility scores for each health state were summarized descriptively for the total population and subgroups based on age, sex, education level, and annual household income. Age and household income were categorized into two groups using the median as a cutoff, and education level was categorized as high for participants who completed college/university or graduate school. Participants who took < 3 min to answer the third training question (wheelchair scenario), took < 6 min to answer TTO questions in the main survey, or showed inconsistency in the health status valuation were considered to not meet the quality standards for valuation. The results, excluding these participants, were summarized as a subpopulation with acceptable valuation quality.

In both the total population and subgroups, the states of participants with a utility score of ≤ − 0.95 were defined as the floor (health state nearly equal to dead), and those with a utility score of ≥ 0.95 were defined as the ceiling (nearly equal to full health); the number of these participants was counted. Utility scores were compared between two health states in all possible combinations using paired *t*-tests. Utility scores between subgroups were analyzed using unpaired *t*-test. The results from each interviewer were compared in each health state by analysis of variance (ANOVA) to detect potential interviewer-related bias.

Furthermore, to confirm the robustness of the difference in utility scores for each health state, we estimated the impact of covariates such as social and clinical characteristics of participants using regression analysis, as described in a previous study^33^. In addition to ordinary least squares, a random-effects maximum likelihood estimator (a mixed-effects model including random effects of participants) and Tobit models accounting for data distribution restricted between − 1 and 1, were used for the estimation. Covariates were selected using the forward-backward stepwise selection method in the ordinary least squares model and applied to the other models as well. The fit of each regression model was evaluated using R-squared values, along with the number and mean absolute error of inconsistent coefficients.

The interview data were anonymized at the study site and statistically analyzed centrally. For all analyses, SAS version 9.4 (SAS Institute, Cary, NC, USA) was used. A p-value of ≤ 0.05 was considered statistically significant.

## Results

### Participants’ characteristics

In total, 107 participants were eligible and took part in the main survey. The mean (standard deviation) age of the participants was 44.5 (14.32) years, and 50.5% were male. These data were comparable to the national averages from the Japanese population census (mean age, 47.7 years; male, 48.6%) (Table 3). Compared with the general population, participants in this study were more likely to have a higher education (high education: 70.1% vs. 27.7%), be employed as regular staff/employees (46.7% vs. 32.9%), be single (43.9% vs. 25.6%), and have a higher annual household income. Most participants had not visited a hospital in the past month (88.8%), which was higher than the national standard (58.3%) (Supplementary Table S1). No participants were undergoing hemodialysis.

**Table 3:** Characteristics of participants.

	Study participants, *n* (%)^a^	General population, %^b^
Total N	107	NA
Age in years, mean (s.d.)	44.5 (14.32)	47.7 (NA)
Sex, male	54 (50.5)	48.6
Highest level education completed		
Graduate school	4 (3.7)	3.0
College/university (bachelor’s degree)	71 (66.4)	24.7
College (associate degree)	9 (8.4)	8.0
Technical school	7 (6.5)	12.3
Senior high school	16 (15.0)	38.4
Junior high school	0 (0.0)	11.6
Employment status		
Employed		
Regular staff/employee	50 (46.7)	32.9
Part-time staff/employee	11 (10.3)	9.4
Marginal part-time worker	6 (5.6)	3.9
Temporary worker from a dispatching office	6 (5.6)	1.4
Contract staff	9 (8.4)	2.7
Commissioned worker	3 (2.8)	1.0
Company executive	1 (0.9)	0.9
Self-employed worker (with employees)	0 (0.0)	3.6
Self-employed worker (without employees)	6 (5.6)	0.9
Helping with the family business	0 (0.0)	0.1
Side job	0 (0.0)	0.8
Not working		
Seeking a job	0 (0.0)	2.9
Attending school	4 (3.7)	5.1
Housekeeping	8 (7.5)	16.4
Others (e.g., retired)	3 (2.8)	14.7
Marital status		
Unmarried	47 (43.9)	25.6
Married	53 (49.5)	54.4
Single (widowed)	0 (0.0)	8.2
Single (divorced)	7 (6.5)	5.0
No answer	0 (0.0)	6.8
Annual household income [million yen]		
< 2	4 (3.7)	19.7
≥ 2 to < 3	9 (8.4)	14.6
≥ 3 to < 4	14 (13.1)	12.6
≥ 4 to < 5	15 (14.0)	10.3
≥ 5 to < 6	10 (9.3)	8.4
≥ 6 to < 7	10 (9.3)	7.3
≥ 7 to < 8	9 (8.4)	6.2
≥ 8 to < 9	7 (6.5)	4.9
≥ 9 to < 10	9 (8.4)	3.6
≥ 10	20 (18.7)	12.4

^a^Unless otherwise noted.

^b^General population values from the official statistics of the Japanese government^25–31^.

NA, Not available; s.d., standard deviation.

### Utility scores

**Fig. 1:**
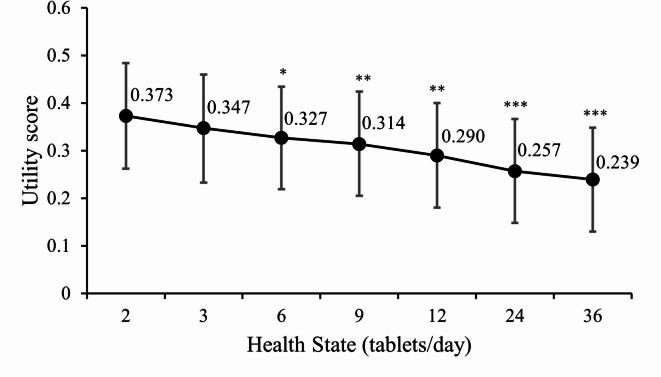
Mean utility scores by health state Created with Excel, PowerPoint, and Photoshop CS2. Mean (95% confidence interval). *p < 0.05, **p < 0.01, and ***p < 0.001 by *t*-test (vs. 2 tablets per day).

Utility scores for each health state are descriptively summarized in Table 4. The utility scores ranged from a minimum of − 1.000 to a maximum of 0.950 across all health states. However, the mean utility score generally decreased as the number of tablets per day increased, with the highest mean utility score (0.373) in the 2-tablet/day health state and the lowest mean utility score (0.239) in the 36-tablet/day health state (Fig. 1). This trend was consistent across all subgroups based on age, sex, education level, annual household income, and participants with acceptable valuation quality (Supplementary Table S2). A nearly equal number of participants across all health states (8–10 participants per group, 7.5–9.3%) scored the health states as floor (utility score ≤ − 0.95). In the 2-tablet/day health state, 4 (3.7%) participants scored it as ceiling (> 0.95), while only 1 (0.9%) participant in each of the 6-, 9-, 12-, 24-, and 36-tablet/day health states scored it as ceiling.

**Table 4:** Utility scores by health state (number of tablets): total population, *N* = 107.

Health state	Mean	s.d.	Median	Q1, Q3	95% CI	Floor^a^ (*n*, %)	Ceiling^a^ (*n*, %)
2 tablets/day	0.373	0.579	0.550	0.200, 0.800	0.262–0.484	9 (8.4)	4 (3.7)
3 tablets/day	0.347	0.591	0.600	0.100, 0.800	0.233–0.460	10 (9.3)	3 (2.8)
6 tablets/day	0.327	0.563	0.400	0.100, 0.750	0.219–0.434	8 (7.5)	1 (0.9)
9 tablets/day	0.314	0.570	0.400	0.100, 0.800	0.205–0.424	9 (8.4)	1 (0.9)
12 tablets/day	0.290	0.575	0.400	0.100, 0.700	0.180–0.400	8 (7.5)	1 (0.9)
24 tablets/day	0.257	0.568	0.300	0.050, 0.700	0.148–0.366	10 (9.3)	1 (0.9)
36 tablets/day	0.239	0.568	0.400	0.100, 0.700	0.130–0.348	10 (9.3)	1 (0.9)

^a^Utility score of ≤ − 0.95 was defined as floor and > 0.95 as ceiling.

CI, Confidence interval; Q1, first quartile; Q3, third quartile; s.d., standard deviation.

Covariate analysis identified age, highest level of education completed, employment status, marital status, annual household income, hospital visits (for conditions such as depression or other mental illnesses, ocular diseases, ear diseases, hypertension, and stiff shoulders), and having an acquaintance undergoing dialysis as the covariates. When these covariates were not included in the model, the random-effects maximum likelihood estimator showed the best fit, with an R-squared value of 0.946, compared with the ordinary least-squares model (R-squared value = 0.006) and the Tobit model (R-squared value = − 0.004). When covariates were included, the random-effects maximum likelihood estimator also showed the best fit, with an R-squared value of 0.946, compared with the ordinary least-squares model showed the best fit (R-squared value = 0.427) and the Tobit model (R-squared value = 0.177). In the model not including the covariate, the regression coefficients for each health state were comparable to the differences in utility scores between health states. It was consistent even when the covariates were included.

### Subgroup analysis

Utility scores for each number of tables per day were compared between subgroups based on age, sex, education level, and annual household income (Supplementary Table S3). Participants who were older (vs. younger), female (vs. male), or had lower education (vs. higher education) tended to assign Lower utility scores, although there were no significant differences between any two subgroup categories across any of the health states. Participants with higher annual household income tended to assign Lower utility scores than participants with lower income, with statistically significant differences observed for the 3-, 6-, 9-, and 36-tablet/day health states. Participants with high valuation quality tended to assign lower utility scores than the total population (Supplementary Table S4).

### Utility differences between two different health States

Utility scores were compared using paired *t*-tests between two health states in all possible combinations (Supplementary Table S5). The difference in utility scores was significant for most comparisons, except between two adjacent health states in the total population.

Interviewer bias.

Given that participants were interviewed by 10 different interviewers, potential bias among interviewers was evaluated. ANOVA revealed no significant interviewer effects, and all interviewers showed a similar pattern in utility distribution: lower utility scores were associated with health states with a higher number of tablets (Supplementary Table S6).

## Discussion

The impact of pill burden on utility scores for health state utility in patients undergoing hemodialysis was estimated using a vignette-based TTO approach in the general population. Our findings demonstrated that utility scores decreased as the number of tablets increased.

Overall, the utility scores estimated by the general population in this study (0.373–0.239) were Lower than those in previous studies: 0.738 by the EQ-5D 5-Level (EQ-5D-5 L) in older patients (≥ 65 years) with ≥ 5 years of hemodialysis vintage^34^, 0.768 by the EQ-5D 3-Level (EQ-5D-3 L) in patients with ≥ 10 years of hemodialysis vintage^35^, and 0.825 in patients undergoing peritoneal dialysis and 0.785 in those undergoing hemodialysis by the EQ-5D-3 L^36^. The EQ-5D and EQ-5D-5 L measure the impact of disease on five QOL dimensions: mobility, self-care, usual activities, pain/discomfort, and anxiety/depression^37^. Adherence related factors, such as pill burden may have a less direct impact on these dimensions. Ahmed et al.^14^ indirectly evaluated low adherence in patients with diabetes through associated increases in pain and anxiety, rather than assessing adherence directly using the EQ-5D. This suggests that the EQ-5D may lack sufficient sensitivity for capturing adherence-related factors. Additionally, discrepancies likely exist between how the general population imagines the health states described in vignettes and how patients with CKD actually feel. In the present study, participants who responded that their health state was nearly equal to death, the floor (utility score of ≤ − 0.95), accounted for 7.5–9.3% of cases with a daily pill burden of 2 to 36 tablets (Table 4). Moreover, 88.8% of the participants did not visit a hospital within 1 month, and only a few had comorbidities or were undergoing medication treatment, which may have affected the low utility scores. Nonetheless, the vignettes were developed with input from patients with CKD undergoing hemodialysis and dialysis experts, making them appropriate for this study.

A previous study evaluating the utility scores of medication burden in the treatment of hepatitis C showed that the utility of taking 7 tablets per day was significantly Lower than that of taking 2 tablets per day, with a mean (standard deviation) difference of 0.01 (0.03)^15^. In the present study, the utility of taking 6 tablets per day was also significantly Lower than that of taking 2 tablets per day, with a mean (standard deviation) difference of 0.046 (0.223). Although both studies used a vignette-based TTO method in the general population, the impact of pill burden on utility scores differed. The greater impact observed in this study is likely due to the influence of water restriction, which is a critical concern for patients undergoing dialysis. In these patients, impaired renal function results in reduced or absent urine production, and inter-dialysis weight gain is associated with a poor prognosis^38^. Therefore, strict water restriction is necessary, and the need to consume water when taking large numbers of tablets may exacerbate the treatment burden.

Conventional phosphate binders, such as sevelamer, often require numerous tablets daily. A previous study showed that taking many tablets could be a mental burden, with many patients expressing a preference for reducing the number of tablets, even to just one^10^. Sucroferric oxyhydroxide was shown to be non-inferior to sevelamer in controlling serum phosphate while substantially reducing the number of tablets required in a phase 3 open-label study^39^. Tenapanor hydrochloride, used to treat hyperphosphatemia without a phosphate-binding property, requires only two tablets per day^40^. In a phase 3 open-label study^41^, the number of tablets was gradually reduced over 52 weeks by replacing phosphate binders with tenapanor hydrochloride while successfully controlling serum phosphate levels. Such drugs may be a promising approach to improving the QOL of patients with CKD undergoing hemodialysis. The vignette-based approach used in this study effectively demonstrated improved utility scores for QOL, with a reduction in the number of tablets. Thus, the vignette-based approach may be useful for assessing QOL changes associated with such treatments, supporting their use in cost-utility analyses.

This study was conducted in Tokyo, Japan. The characteristics of the participants differed somewhat from the general Japanese population, and our subpopulation comparisons indicated that these differences might affect utility valuation. Therefore, it may not fully represent the general Japanese population, although the sample size (*n* = 107) was considered sufficient for generalizability.

The present study was conducted based on the Guideline for Preparing Cost-Effectiveness Evaluation to the Central Social Insurance Medical Council in Japan^11^. This guideline emphasizes that QOL score should reflect the values of the general population, and recommends that direct methods such as TTO be used in the present study. Therefore, in this study, members of the general population were enrolled as study participants, and dialysis patients only participated in the pilot study to create easy-to-understand vignettes.

A major strength of this study is that it is the first to assess health utilities related to pill burden in hemodialysis therapy, supported by a large-scale cohort of participants. However, this study has limitations associated with the vignette-based method. The imagined health states from the vignette description do not directly represent the actual experiences of patients with CKD. The vignettes cannot capture all details of the health states, such as itching and pain, or every possible experience, such as mobility limitations and the actual burden of commuting to the hospital or clinic, which may be important for QOL in some patients. Additionally, the methods are not standardized, which makes comparison with other studies challenging. The health utility scores observed in this study were generally lower than those reported for patients undergoing dialysis using the EQ-5D^34–36^. In the present study, we evaluated utility scores using vignettes to elicit recollections of hemodialysis conditions in a general population and did not evaluate utility scores of EQ-5D. The utility scores obtained in this study cannot be compared with EQ-5D scores. Therefore, changes in health utilities due to differences in pill burden may be more suitable for relative comparisons rather than as absolute values.

## Conclusions

Using a vignette-based TTO approach in the general population, we estimated QOL in patients with CKD across seven different health states and demonstrated the association between pill burden and QOL. The results suggest that this vignette-based approach is feasible for calculating quality-adjusted life years in cost-utility analyses for treatment in patients with CKD undergoing dialysis. Moreover, optimizing prescriptions and simplifying medication regimens to reduce pill burden may help improve QOL in patients undergoing hemodialysis. The results of this study are expected to be translated into healthcare policy and reimbursement decisions, thereby enabling a greater impact on a variety of value assessments.

## Supplementary Information

Below is the link to the electronic supplementary material.

Supplementary Material 1

## Data Availability

No datasets were generated or analysed during the current study.
