# Supplementary material for: Estimating the impact of pill burden on health utilities in hemodialysis therapy
Source: Sci Rep. 2025 Oct 9;15:35315. doi: 10.1038/s41598-025-19346-3 (PMC12511388; doi:10.1038/s41598-025-19346-3)
Supplement: Supplementary file 1 — Supplementary Material 1 [file 41598_2025_19346_MOESM1_ESM.pdf]

Supplementary Information

Estimating the impact of pill burden on health utilities in hemodialysis therapy

Scientific Reports

Hiroo Shimoda, Masatomo Taniguchi, Suguru Yamamoto, Ataru Igarashi, Shin Tokunaga, Keigo Hanada, Naoki Tashiro, Tatsunori Murata, Shinji Asada*

*Corresponding author

Shinji Asada, MPH

Medical Affairs Department, Kyowa Kirin Co., Ltd., Chiyoda-ku, Tokyo, Japan

E-mail: shinji.asada.qd@kyowakirin.com

TEL: +81-3-5205-7200

FAX: +81-3-5205-7182

Supplementary Figure S1. the process of implementing the TTO method

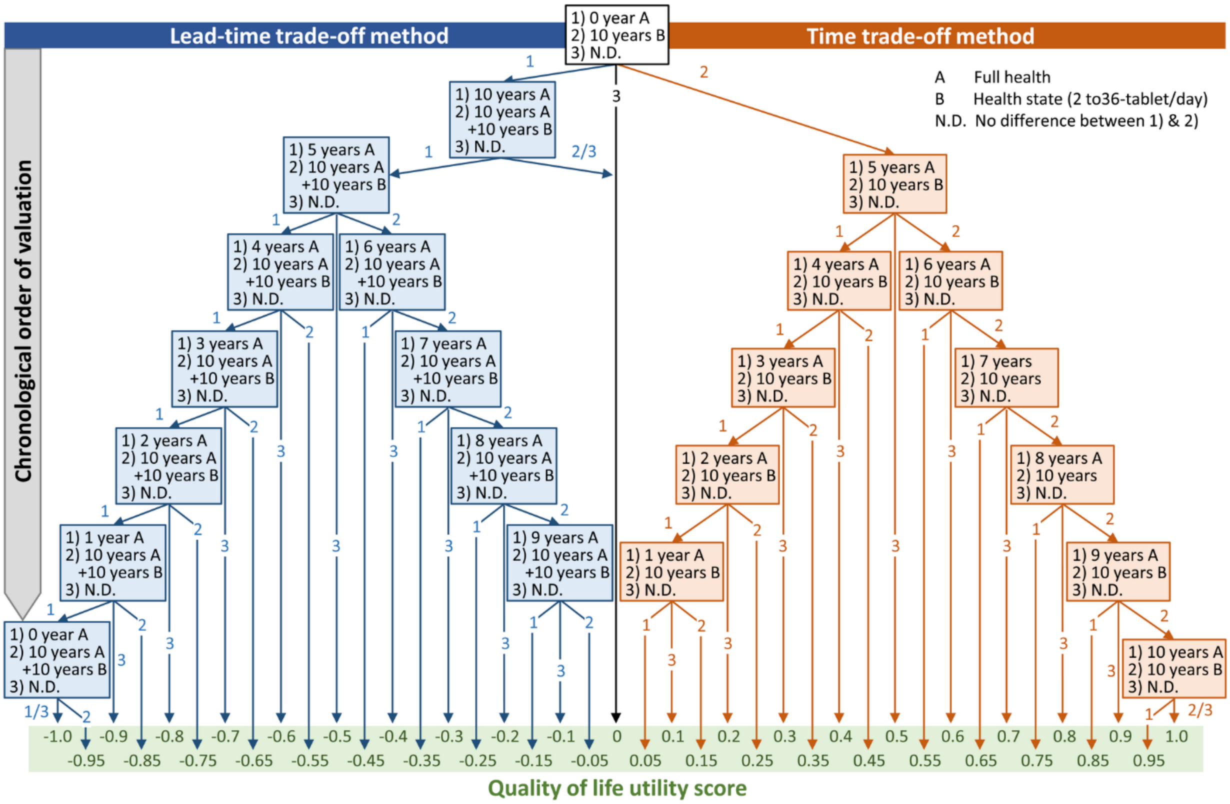

Supplementary Table S1. Clinical status of participants

	Study participants n (%)[a]	General population[b] %
Total N	107	NA
No hospital visits for the last 1 month	95 (88.8)	58.3
Hospital visits for the last 1 month		
Endocrine and metabolic disorders		
Diabetes mellitus	0 (0.0)	13.3
Obesity	0 (0.0)	1.3
Dyslipidemia (e.g., hypercholesterolemia)	1 (0.9)	15.8
Thyroid disease	0 (0.0)	3.6
Psychiatric or neurologic disorders		
Depression or other mental illness	1 (0.9)	5.2
Dementia	0 (0.0)	1.6
Parkinson's disease	0 (0.0)	0.6
Other neurological disease (e.g., neuralgia, palsy)	0 (0.0)	1.6
Ocular diseases	1 (0.9)	13.8
Ear diseases	1 (0.9)	2.5
Cardiovascular diseases		

	Study participants n (%)[a]	General population[b] %
Total N	107	NA
Hypertension	1 (0.9)	33.8
Stroke (e.g., cerebral hemorrhage, cerebral infarction)	0 (0.0)	2.6
Angina pectoris, myocardial infarction	0 (0.0)	4.3
Other cardiovascular diseases	0 (0.0)	4.8
Respiratory diseases		
Acute nasopharyngitis (common cold)	0 (0.0)	0.4
Allergic rhinitis	1 (0.9)	5.7
Chronic obstructive pulmonary disease	0 (0.0)	0.4
Asthma	0 (0.0)	3.3
Other respiratory diseases	0 (0.0)	2.7
Digestive diseases		
Stomach/duodenum diseases	0 (0.0)	3.4
Liver/gallbladder diseases	0 (0.0)	2.0
Other digestive diseases	1 (0.9)	3.4
Dental diseases	1 (0.9)	12.6
Skin diseases		
Atopic dermatitis	1 (0.9)	2.6

	Study participants n (%)[a]	General population[b] %
Total N	107	NA
Other skin diseases	1 (0.9)	4.8
Musculoskeletal diseases		
Gout	0 (0.0)	2.6
Rheumatoid arthritis	0 (0.0)	1.9
Joint disorders	0 (0.0)	5.0
Stiff shoulders	2 (1.9)	5.5
Lower back pain	0 (0.0)	11.5
Osteoporosis	0 (0.0)	5.0
Urinary tract or genital diseases		
Kidney diseases	0 (0.0)	2.7
Prostatic hyperplasia	0 (0.0)	3.4
Menopausal/post-menopausal disorders	0 (0.0)	0.6
Injury		
Fracture	0 (0.0)	1.7
Injury other than fracture/burn	0 (0.0)	1.4
Anemia/blood diseases	0 (0.0)	1.6
Malignant neoplasm (cancer)	0 (0.0)	2.6

	Study participants n (%)[a]	General population[b] %
Total N	107	NA
Pregnancy/puerperia (e.g., threatened miscarriage, placenta previa)	0 (0.0)	0.2
Infertility	0 (0.0)	0.2
Others	4 (3.7)	6.4
Unknown	0 (0.0)	0.3
Under treatment		
Chemotherapy	0 (0.0)	NA
Dialysis	0 (0.0)	NA
Rehabilitation	0 (0.0)	NA
None of the above	107 (100.0)	NA
Any acquaintance under treatment		
Chemotherapy	24 (22.4)	NA
Dialysis	19 (17.8)	NA
Rehabilitation	7 (6.5)	NA
None of the above/unknown	69(64.5)	NA
Number of tablets taken per day		
Mean (s.d.)	0.3 (1.26)	NA

	Study participants<br>n (%)[a]	General population[b]<br>%
Total N	107	NA
Frequency of tablet intake [times/day]		
Mean (s.d.)	0.2 (0.65)	NA

[a]Unless otherwise noted

[b]General population values from the official statistics of the Japanese government [エラー! 参照元が見つかりません。]

NA, Not available; s.d., standard deviation

Supplementary Table S2. Utility scores by health state (number of tablets): subpopulation

Health state	Mean	s.d.	Median	Q1, Q3	95% CI	Floor[a] (n, %)	Ceiling[b] (n, %)
Age, older half[c], n = 55							
2 tablets/day	0.307	0.594	0.400	0.150, 0.800	0.147–0.468	5 (9.1)	2 (3.6)
3 tablets/day	0.282	0.612	0.400	0.000, 0.750	0.116–0.447	7 (12.7)	2 (3.6)
6 tablets/day	0.240	0.579	0.400	0.000, 0.700	0.083–0.397	5 (9.1)	1 (1.8)
9 tablets/day	0.263	0.596	0.400	0.100, 0.750	0.102–0.424	6 (10.9)	1 (1.8)
12 tablets/day	0.203	0.597	0.350	0.000, 0.700	0.041–0.364	5 (9.1)	1 (1.8)
24 tablets/day	0.195	0.566	0.300	0.000, 0.650	0.042–0.348	6 (10.9)	1 (1.8)
36 tablets/day	0.175	0.589	0.350	0.000, 0.600	0.015–0.334	7 (12.7)	1 (1.8)
Age, younger half[d], n = 52							
2 tablets/day	0.442	0.560	0.700	0.300, 0.850	0.286–0.598	4 (7.7)	2 (3.8)
3 tablets/day	0.415	0.566	0.700	0.300, 0.800	0.258–0.573	3 (5.8)	1 (1.9)
6 tablets/day	0.418	0.535	0.675	0.250, 0.800	0.269–0.567	3 (5.8)	0 (0.0)
9 tablets/day	0.369	0.543	0.575	0.175, 0.800	0.218–0.520	3 (5.8)	0 (0.0)
12 tablets/day	0.383	0.540	0.625	0.200, 0.800	0.232–0.533	3 (5.8)	0 (0.0)
24 tablets/day	0.322	0.569	0.600	0.100, 0.775	0.164–0.481	4 (7.7)	0 (0.0)
36 tablets/day	0.307	0.542	0.450	0.100, 0.700	0.156–0.458	3 (5.8)	0 (0.0)
	Male, n = 54						
2 tablets/day	0.394	0.538	0.475	0.150, 0.800	0.247–0.540	3 (5.6)	3 (5.6)

Health state	Mean	s.d.	Median	Q1, Q3	95% CI	Floor[a] (n, %)	Ceiling[b] (n, %)
3 tablets/day	0.368	0.576	0.575	0.100, 0.800	0.210–0.525	4 (7.4)	3 (5.6)
6 tablets/day	0.371	0.533	0.525	0.150, 0.800	0.226–0.517	3 (5.6)	1 (1.9)
9 tablets/day	0.345	0.543	0.425	0.100, 0.800	0.197–0.494	4 (7.4)	1 (1.9)
12 tablets/day	0.322	0.548	0.325	0.100, 0.750	0.173–0.472	3 (5.6)	1 (1.9)
24 tablets/day	0.291	0.528	0.300	0.100, 0.700	0.147–0.435	3 (5.6)	1 (1.9)
36 tablets/day	0.262	0.540	0.325	0.100, 0.700	0.115–0.409	3 (5.6)	1 (1.9)
Female, n = 53							
2 tablets/day	0.352	0.623	0.600	0.300, 0.800	0.180–0.523	6 (11.3)	1 (1.9)
3 tablets/day	0.325	0.611	0.600	0.200, 0.750	0.157–0.494	6 (11.3)	0 (0.0)
6 tablets/day	0.281	0.593	0.400	0.000, 0.700	0.118–0.445	5 (9.4)	0 (0.0)
9 tablets/day	0.283	0.601	0.400	0.100, 0.800	0.117–0.449	5 (9.4)	0 (0.0)
12 tablets/day	0.258	0.604	0.400	0.000, 0.700	0.091–0.424	5 (9.4)	0 (0.0)
24 tablets/day	0.223	0.610	0.350	0.000, 0.700	0.055–0.391	7 (13.2)	0 (0.0)
36 tablets/day	0.215	0.599	0.400	0.100, 0.700	0.050–0.380	7 (13.2)	0 (0.0)
Education, higher half[e], n = 75							
2 tablets/day	0.393	0.596	0.650	0.200, 0.800	0.256–0.530	6 (8.0)	4 (5.3)
3 tablets/day	0.375	0.590	0.650	0.200, 0.800	0.240–0.511	6 (8.0)	3 (4.0)
6 tablets/day	0.365	0.555	0.550	0.150, 0.800	0.237–0.492	4 (5.3)	1 (1.3)
9 tablets/day	0.345	0.568	0.600	0.100, 0.800	0.215–0.476	5 (6.7)	1 (1.3)

Health state	Mean	s.d.	Median	Q1, Q3	95% CI	Floor[a] (n, %)	Ceiling[b] (n, %)
12 tablets/day	0.320	0.581	0.500	0.100, 0.800	0.186–0.454	4 (5.3)	1 (1.3)
24 tablets/day	0.279	0.575	0.500	0.100, 0.700	0.146–0.411	6 (8.0)	1 (1.3)
36 tablets/day	0.251	0.579	0.400	0.100, 0.700	0.117–0.384	7 (9.3)	1 (1.3)
Education, lower half[f], n = 32							
2 tablets/day	0.327	0.544	0.400	0.250, 0.700	0.130–0.523	3 (9.4)	0 (0.0)
3 tablets/day	0.280	0.597	0.400	0.050, 0.700	0.064–0.495	4 (12.5)	0 (0.0)
6 tablets/day	0.238	0.579	0.325	0.050, 0.700	0.029–0.446	4 (12.5)	0 (0.0)
9 tablets/day	0.242	0.577	0.375	0.100, 0.700	0.034–0.450	4 (12.5)	0 (0.0)
12 tablets/day	0.220	0.562	0.325	0.050, 0.675	0.018–0.423	4 (12.5)	0 (0.0)
24 tablets/day	0.206	0.559	0.300	0.025, 0.600	0.005–0.408	4 (12.5)	0 (0.0)
36 tablets/day	0.211	0.547	0.375	0.075, 0.600	0.014–0.408	3 (9.4)	0 (0.0)
Annual household income, higher half[g], n = 55							
2 tablets/day	0.298	0.580	0.350	0.150, 0.800	0.141–0.455	4 (7.3)	1 (1.8)
3 tablets/day	0.215	0.632	0.350	0.000, 0.700	0.044–0.385	7 (12.7)	0 (0.0)
6 tablets/day	0.209	0.590	0.300	0.000, 0.700	0.050–0.368	5 (9.1)	0 (0.0)
9 tablets/day	0.209	0.601	0.300	0.000, 0.700	0.046–0.372	6 (10.9)	0 (0.0)
12 tablets/day	0.194	0.601	0.300	0.000, 0.700	0.031–0.356	5 (9.1)	0 (0.0)
24 tablets/day	0.167	0.577	0.250	0.000, 0.650	0.011–0.323	6 (10.9)	0 (0.0)
36 tablets/day	0.123	0.597	0.200	0.000, 0.600	−0.039–0.284	6 (10.9)	0 (0.0)

Health state	Mean	s.d.	Median	Q1, Q3	95% CI	Floor[a] (n, %)	Ceiling[b] (n, %)
Annual household income, lower half[h], n = 52							
2 tablets/day	0.452	0.573	0.700	0.300, 0.800	0.292–0.611	5 (9.6)	3 (5.8)
3 tablets/day	0.487	0.515	0.700	0.300, 0.800	0.343–0.630	3 (5.8)	3 (5.8)
6 tablets/day	0.451	0.509	0.700	0.300, 0.800	0.309–0.593	3 (5.8)	1 (1.9)
9 tablets/day	0.426	0.518	0.625	0.300, 0.800	0.282–0.570	3 (5.8)	1 (1.9)
12 tablets/day	0.392	0.533	0.600	0.300, 0.800	0.244–0.541	3 (5.8)	1 (1.9)
24 tablets/day	0.352	0.549	0.600	0.150, 0.775	0.199–0.505	4 (7.7)	1 (1.9)
36 tablets/day	0.362	0.513	0.550	0.200, 0.700	0.219–0.504	4 (7.7)	1 (1.9)
Fulfilling quality standard for valuation[i], n = 53							
2 tablets/day	0.271	0.664	0.350	0.100, 0.800	0.088–0.454	7 (13.2)	2 (3.8)
3 tablets/day	0.244	0.660	0.350	0.000, 0.800	0.063–0.426	7 (13.2)	2 (3.8)
6 tablets/day	0.241	0.661	0.350	0.000, 0.800	0.058–0.423	7 (13.2)	1 (1.9)
9 tablets/day	0.233	0.656	0.350	0.000, 0.800	0.052–0.414	7 (13.2)	1 (1.9)
12 tablets/day	0.233	0.656	0.350	0.000, 0.800	0.052–0.414	7 (13.2)	1 (1.9)
24 tablets/day	0.211	0.647	0.300	0.000, 0.700	0.033–0.390	7 (13.2)	1 (1.9)
36 tablets/day	0.195	0.645	0.300	0.000, 0.700	0.018–0.373	8 (15.1)	1 (1.9)

[a]Utility score of ≤−0.95 was defined as floor

[b]Utility score of >0.95 was defined as ceiling

[c]Older half was defined as ≥44.0 years (median age)

[d]Younger half was defined as <44.0 years (median age)

[e]Higher half in education was defined as graduate school or college/university

[f]Lower half in education was defined as junior college, professional training college, senior high school, or junior high school

[g]Higher half in annual household income was defined as ≥6 million yen

[h]Lower half in annual household income was defined as <6 million yen

[i]Participants who required <3 minutes of explanation for the third training question (wheelchair as an example), presented obvious inconsistency in lead- TTO or TTO valuations, or answered health state questions in <6 minutes were considered less likely to meet the valuation quality standard and were therefore removed from the data set.

CI, Confidence interval; Q1, first quartile; Q3, third quartile; s.d., standard deviation; TTO, time trade-off

Supplementary Table S3. Subgroup analysis

Age									
	Older half[a], n = 55		Younger half[b], n = 52		Difference				Cohen's d
Health state	Mean	s.d.	Mean	s.d.	Mean	s.d.	*t*	*p*	
2 tablets/day	0.307	0.594	0.442	0.560	−0.135	0.578	−1.211	0.229	0.234
3 tablets/day	0.282	0.612	0.415	0.566	−0.134	0.590	−1.173	0.244	0.227
6 tablets/day	0.240	0.579	0.418	0.535	−0.178	0.558	−1.655	0.101	0.320
9 tablets/day	0.263	0.596	0.369	0.543	−0.107	0.571	−0.968	0.335	0.187
12 tablets/day	0.203	0.597	0.383	0.540	−0.180	0.570	−1.636	0.105	0.316
24 tablets/day	0.195	0.566	0.322	0.569	−0.127	0.568	−1.153	0.251	0.223
36 tablets/day	0.175	0.589	0.307	0.542	−0.132	0.566	−1.209	0.229	0.234

Sex									
	Male, n = 54		Female, n = 53		Difference				Cohen's d
Health state	Mean	s.d.	Mean	s.d.	Mean	s.d.	*t*	*p*	
2 tablets/day	0.394	0.538	0.352	0.623	0.042	0.581	0.370	0.712	0.072
3 tablets/day	0.368	0.576	0.325	0.611	0.042	0.594	0.367	0.715	0.071
6 tablets/day	0.371	0.533	0.281	0.593	0.090	0.564	0.827	0.410	0.160
9 tablets/day	0.345	0.543	0.283	0.601	0.062	0.572	0.563	0.575	0.109
12 tablets/day	0.322	0.548	0.258	0.604	0.065	0.577	0.580	0.563	0.112
24 tablets/day	0.291	0.528	0.223	0.610	0.068	0.570	0.617	0.539	0.119

36 tablets/day	0.262	0.540	0.215	0.599	0.047	0.570	0.426	0.671	0.082
Education									
	Higher half[c], n = 75		Lower half[d], n = 32		Difference				Cohen's d
Health state	Mean	s.d.	Mean	s.d.	Mean	s.d.	*t*	*p*	
2 tablets/day	0.393	0.596	0.327	0.544	0.066	0.581	0.559	0.578	0.116
3 tablets/day	0.375	0.590	0.280	0.597	0.096	0.592	0.761	0.450	0.161
6 tablets/day	0.365	0.555	0.238	0.579	0.127	0.562	1.053	0.297	0.224
9 tablets/day	0.345	0.568	0.242	0.577	0.103	0.571	0.850	0.399	0.180
12 tablets/day	0.320	0.581	0.220	0.562	0.100	0.576	0.831	0.409	0.174
24 tablets/day	0.279	0.575	0.206	0.559	0.072	0.570	0.608	0.545	0.128
36 tablets/day	0.251	0.579	0.211	0.547	0.040	0.570	0.338	0.737	0.071
Annual household income									
	Higher half[e], n = 55		Lower half[f], n = 52		Difference				Cohen's d
Health state	Mean	s.d.	Mean	s.d.	Mean	s.d.	*t*	*p*	
2 tablets/day	0.298	0.580	0.452	0.573	−0.154	0.576	−1.379	0.171	0.267
3 tablets/day	0.215	0.632	0.487	0.515	−0.272	0.578	−2.447	0.016	0.472
6 tablets/day	0.209	0.590	0.451	0.509	−0.242	0.552	−2.274	0.025	0.439
9 tablets/day	0.209	0.601	0.426	0.518	−0.217	0.563	−2.002	0.048	0.386
12 tablets/day	0.194	0.601	0.392	0.533	−0.199	0.569	−1.812	0.073	0.350
24 tablets/day	0.167	0.577	0.352	0.549	−0.185	0.564	−1.696	0.093	0.328

36 tablets/day	0.123	0.597	0.362	0.513	−0.239	0.558	−2.224	0.028		0.429

[a]Older half was defined as ≥44.0 years (median age)

[b]Younger half was defined as <44.0 years (median age)

[c]Higher half in education was defined as graduate school or college/university

[d]Lower half in education was defined as junior college, professional training college, senior high school, or junior high school

[e]Higher half in annual household income was defined as ≥6 million yen

[f]Lower half in annual household income was defined as <6 million yen

s.d., Standard deviation

Supplementary Table S4. Subgroup analysis: participants with acceptable valuation quality

	Total population, n = 107		Participants with acceptable valuation quality, n = 53	
Health state	Mean	s.d.	Mean	s.d.
2 tablets/day	0.373	0.579	0.271	0.664
3 tablets/day	0.347	0.591	0.244	0.660
6 tablets/day	0.327	0.563	0.241	0.661
9 tablets/day	0.314	0.570	0.233	0.656
12 tablets/day	0.290	0.575	0.233	0.656
24 tablets/day	0.257	0.568	0.211	0.647
36 tablets/day	0.239	0.568	0.195	0.645

s.d., Standard deviation

Supplementary Table S5. Comparisons of utility scores among health state groups (number of tablets): total population, N = 107

	Utility score		Difference in utility score (vs. reference)							
Health state	Mean	s.d.	Mean	s.d.	Median	Q1, Q3	Min, Max	95% CI	*t* paired test	*p*
2 tablets/day (reference)	0.373	0.579								
3 tablets/day	0.347	0.591	0.026	0.241	0.000	0.000, 0.100	−1.500, 1.200	−0.020–0.072	1.125	0.263
6 tablets/day	0.327	0.563	0.046	0.223	0.000	0.000, 0.100	−1.500, 1.000	0.004–0.089	2.148	0.034
9 tablets/day	0.314	0.570	0.058	0.220	0.000	0.000, 0.100	−1.400, 1.000	0.016–0.101	2.750	0.007
12 tablets/day	0.290	0.575	0.083	0.279	0.000	0.000, 0.100	−1.700, 1.150	0.029–0.136	3.068	0.003
24 tablets/day	0.257	0.568	0.116	0.267	0.100	0.000, 0.150	−1.600, 1.000	0.065–0.167	4.497	<0.001
36 tablets/day	0.239	0.568	0.134	0.266	0.100	0.000, 0.200	−1.300, 1.100	0.083–0.185	5.220	<0.001
3 tablets/day (reference)	0.347	0.591								
6 tablets/day	0.327	0.563	0.020	0.143	0.000	0.000, 0.050	−1.100, 0.350	−0.007–0.047	1.456	0.148
9 tablets/day	0.314	0.570	0.032	0.179	0.000	0.000, 0.100	−1.100, 1.100	−0.002–0.067	1.866	0.065
12 tablets/day	0.290	0.575	0.057	0.222	0.000	0.000, 0.050	−1.100, 1.100	0.014–0.099	2.633	0.010
24 tablets/day	0.257	0.568	0.090	0.225	0.000	0.000, 0.100	−0.900, 1.300	0.047–0.133	4.133	<0.001
36 tablets/day	0.239	0.568	0.108	0.186	0.100	0.000, 0.200	−0.650, 0.700	0.072–0.144	5.992	<0.001

	Utility score		Difference in utility score (vs. reference)							
Health state	Mean	s.d.	Mean	s.d.	Median	Q1, Q3	Min, Max	95% CI	*t* paired test	*p*
6 tablets/day (reference)	0.327	0.563								
9 tablets/day	0.314	0.570	0.012	0.141	0.000	0.000, 0.000	−0.400, 1.000	−0.015–0.039	0.892	0.374
12 tablets/day	0.290	0.575	0.036	0.169	0.000	0.000, 0.000	−0.300, 1.000	0.004–0.069	2.225	0.028
24 tablets/day	0.257	0.568	0.070	0.181	0.000	0.000, 0.100	−0.200, 1.200	0.035–0.104	3.970	<0.001
36 tablets/day	0.239	0.568	0.088	0.186	0.050	0.000, 0.100	−0.650, 1.000	0.052–0.124	4.881	<0.001
9 tablets/day (reference)	0.314	0.570								
12 tablets/day	0.290	0.575	0.024	0.132	0.000	0.000, 0.000	−0.300, 0.850	−0.001–0.050	1.904	0.060
24 tablets/day	0.257	0.568	0.057	0.137	0.000	0.000, 0.100	−0.200, 0.850	0.031–0.084	4.351	<0.001
36 tablets/day	0.239	0.568	0.076	0.201	0.000	0.000, 0.100	−0.600, 1.000	0.037–0.114	3.889	<0.001
12 tablets/day (reference)	0.290	0.575								
24 tablets/day	0.257	0.568	0.033	0.177	0.000	0.000, 0.100	−0.850, 0.750	−0.001–0.067	1.941	0.055
36 tablets/day	0.239	0.568	0.051	0.214	0.000	0.000, 0.100	−0.850, 1.000	0.010–0.092	2.483	0.015

	Utility score		Difference in utility score (vs. reference)							
Health state	Mean	s.d.	Mean	s.d.	Median	Q1, Q3	Min, Max	95% CI	*t*<br>paired test	*p*
24 tablets/day (reference)	0.257	0.568								
36 tablets/day	0.239	0.568	0.018	0.194	0.000	0.000, 0.050	−0.800, 0.800	−0.019–0.055	0.973	0.333

CI, Confidence interval; Q1, first quartile; Q3, third quartile; s.d., standard deviation

Supplementary Table S6. Utility scores for quality of life by interviewers

Interviewer	Number of analyzed participants, n (%)	Utility score, mean (s.d.)	*p*
	Health state: 2 tablets/day		
All interviewers	107	0.373 (0.579)	–
No. 1	11 (10.3)	0.509 (0.530)	0.784
No. 2	11 (10.3)	0.405 (0.591)	
No. 3	11 (10.3)	0.455 (0.541)	
No. 4	10 (9.3)	0.165 (0.620)	
No. 5	11 (10.3)	0.505 (0.559)	
No. 6	11 (10.3)	0.527 (0.434)	
No. 7	10 (9.3)	0.430 (0.564)	
No. 8	10 (9.3)	0.320 (0.577)	
No. 9	11 (10.3)	0.195 (0.813)	
No. 10	11 (10.3)	0.200 (0.588)	
	Health state: 3 tablets/day		
All interviewers	107	0.347 (0.591)	–
No. 1	11 (10.3)	0.482 (0.538)	0.699
No. 2	11 (10.3)	0.432 (0.609)	
No. 3	11 (10.3)	0.427 (0.548)	
No. 4	10 (9.3)	0.135 (0.564)	
No. 5	11 (10.3)	0.423 (0.706)	
No. 6	11 (10.3)	0.532 (0.456)	
No. 7	10 (9.3)	0.450 (0.576)	
No. 8	10 (9.3)	0.265 (0.549)	
No. 9	11 (10.3)	0.195 (0.740)	
No. 10	11 (10.3)	0.109 (0.635)	

Interviewer	Number of analyzed participants, n (%)	Utility score, mean (s.d.)	*p*
	Health state: 6 tablets/day		
All interviewers	107	0.327 (0.563)	–
No. 1	11 (10.3)	0.473 (0.516)	0.845
No. 2	11 (10.3)	0.377 (0.590)	
No. 3	11 (10.3)	0.377 (0.536)	
No. 4	10 (9.3)	0.230 (0.422)	
No. 5	11 (10.3)	0.391 (0.698)	
No. 6	11 (10.3)	0.441 (0.472)	
No. 7	10 (9.3)	0.435 (0.568)	
No. 8	10 (9.3)	0.270 (0.551)	
No. 9	11 (10.3)	0.182 (0.717)	
No. 10	11 (10.3)	0.086 (0.592)	
	Health state: 9 tablets/day		
All interviewers	107	0.314 (0.570)	–
No. 1	11 (10.3)	0.473 (0.506)	0.860
No. 2	11 (10.3)	0.250 (0.588)	
No. 3	11 (10.3)	0.391 (0.526)	
No. 4	10 (9.3)	0.245 (0.421)	
No. 5	11 (10.3)	0.436 (0.713)	
No. 6	11 (10.3)	0.432 (0.472)	
No. 7	10 (9.3)	0.380 (0.569)	
No. 8	10 (9.3)	0.265 (0.559)	
No. 9	11 (10.3)	0.177 (0.740)	
No. 10	11 (10.3)	0.091 (0.633)	
	Health state: 12 tablets/day		
All interviewers	107	0.290 (0.575)	–

Interviewer	Number of analyzed participants, n (%)	Utility score, mean (s.d.)	*p*
No. 1	11 (10.3)	0.455 (0.507)	0.773
No. 2	11 (10.3)	0.132 (0.627)	
No. 3	11 (10.3)	0.414 (0.538)	
No. 4	10 (9.3)	0.255 (0.447)	
No. 5	11 (10.3)	0.405 (0.700)	
No. 6	11 (10.3)	0.386 (0.450)	
No. 7	10 (9.3)	0.380 (0.555)	
No. 8	10 (9.3)	0.265 (0.549)	
No. 9	11 (10.3)	0.173 (0.719)	
No. 10	11 (10.3)	0.041 (0.657)	
	Health state: 24 tablets/day		
All interviewers	107	0.257 (0.568)	–
No. 1	11 (10.3)	0.409 (0.522)	0.780
No. 2	11 (10.3)	0.145 (0.572)	
No. 3	11 (10.3)	0.309 (0.513)	
No. 4	10 (9.3)	0.220 (0.472)	
No. 5	11 (10.3)	0.386 (0.694)	
No. 6	11 (10.3)	0.391 (0.459)	
No. 7	10 (9.3)	0.360 (0.546)	
No. 8	10 (9.3)	0.235 (0.539)	
No. 9	11 (10.3)	0.114 (0.755)	
No. 10	11 (10.3)	0.005 (0.619)	
	Health state: 36 tablets/day		
All interviewers	107	0.239 (0.568)	–
No. 1	11 (10.3)	0.373 (0.512)	0.779
No. 2	11 (10.3)	0.264 (0.526)	

Interviewer	Number of analyzed participants, n (%)	Utility score, mean (s.d.)	*p*
No. 3	11 (10.3)	0.227 (0.549)	
No. 4	10 (9.3)	0.145 (0.612)	
No. 5	11 (10.3)	0.368 (0.664)	
No. 6	11 (10.3)	0.359 (0.453)	
No. 7	10 (9.3)	0.350 (0.548)	
No. 8	10 (9.3)	0.255 (0.536)	
No. 9	11 (10.3)	0.086 (0.734)	
No. 10	11 (10.3)	−0.036 (0.584)	

s.d., Standard deviation
